# Knowledge, attitudes and practices of AIDS associated malignancies among people living with HIV in Nigeria

**DOI:** 10.1186/1750-9378-7-28

**Published:** 2012-10-25

**Authors:** Elima Jedy-Agba, Clement Adebamowo

**Affiliations:** 1 Office of Strategic Information, Training and Research, Institute of Human Virology, Nigeria, 252 Herbert Macaulay Way, Central Business District, Abuja, Federal Capital Territory, Nigeria; 2 Department of Epidemiology and Public Health, and Institute of Human Virology, University of Maryland, 725 W. Lombard St, Baltimore, MD, 21201, USA

**Keywords:** Knowledge, Attitudes, Practices (KAP), People living with HIV (PLHIV), HIV-associated cancers, Cancer screening

## Abstract

**Introduction:**

The epidemic of HIV in sub-Saharan Africa varies significantly across countries in the region with high prevalence in Southern Africa and Nigeria. Cancer is increasingly identified as a complication of HIV infection with higher incidence and mortality in this group than in the general population. Without cancer prevention strategies, improved cancer treatment alone would be an insufficient response to this increasing burden among people living with HIV (PLHIV). Although previous studies have noted low levels of awareness of cancers in sub-Saharan Africa none has examined the knowledge and perceptions of cancer among people living with HIV/AIDS.

**Methods:**

Focus group discussions (FGD) and Key Informant Interviews (KII) were carried out in 4 high volume tertiary care institutions that offer HIV care and treatment in Nigeria. FGD and KII assessed participants’ knowledge of cancer, attitudes towards cancer risk and cancer screening practices.

**Results:**

The mean age (SD) of the FGD participants was 38 (2.8) years. Most participants had heard about cancer and considered it a fatal disease but displayed poor knowledge of the causes of cancer in general and of AIDs associated cancers in particular. PLHIV in Nigeria expressed fear, denial and disbelief about their perceived cancer risk. Some of the participants had heard about cancer screening but very few participants had ever been screened.

**Conclusion:**

Our findings of poor knowledge of cancer among PLHIV in Nigeria indicate the need for health care providers and the government to intervene by developing primary cancer prevention strategies for this population.

## Introduction

Sub-Saharan Africa accounts for 68% of all HIV infected persons worldwide (22.9 million out of 34 million) and 75% of AIDS-related deaths (1.2 million out of 1.8 million) [1]. The epidemic in sub-Saharan Africa varies significantly across the continent with high prevalence in Southern Africa and Nigeria [2]. In Nigeria, the sero-prevalence of HIV among adults aged 15–49 years was 4.1% in 2010 [3].

HIV infection is associated with several co-morbidities including opportunistic infections, (e.g. tuberculosis) and cancers that are responsible for AIDS associated mortality [4,5]. In developed countries where the HIV epidemic has matured and most people living with HIV (PLHIV) are on anti-retroviral therapy, cancer is now responsible for at least a third of all mortality [6]. The incidence of cancer among PLHIV in developing countries too has increased but there is insufficient data on actual incidence and prevalence [7,8].

Cancer is a major source of morbidity and mortality worldwide. In 2008, there were 12.7 million new cases and 7.6 million cancer-related deaths [9]. Projections show that by 2030, cancer will cause 17 million deaths with 70% of these expected to occur in developing countries [10]. Infections including HIV, Hepatitis B and C virus infections and Human papilloma virus infections account for about 30% of incident cancers in developing countries in contrast with developed countries where it accounts for about 5%. HIV has emerged as one of the major infectious risk factors being associated with cancers such as – Kaposi Sarcoma, Non-Hodgkin’s Lymphoma (NHL) and cervical cancer (CC) collectively referred to as AIDS Defining Cancers and other cancers like Lung Cancer, Anal Cancer and Cancer of the Conjunctiva [11].

In many parts of Africa, HIV treatment and prevention is supported by the President’s Emergency Plan for AIDS relief (PEPFAR) and this has led to significant improvement in the overall morbidity and mortality of HIV. As a result of this intervention, survival of PLHIV has increased and the incidence of some AIDS Defining Cancers like NHL and KS have begun to reduce while the incidence of Non-AIDS Defining Cancers (NADCs) are now increasing [12,13]. Given that cancers in resource constrained settings tend to present at advanced stages with limited treatment options [5], preventive and early detection measures such as health education and screening are necessary as part of a comprehensive approach to improved management of people living with HIV [14].

Previous studies have noted low levels of awareness of cancers in sub-Saharan Africa [15,16] and high levels of patronage of alternate medical practitioners [17]. They also identified the need to provide contextual and culturally sensitive health education [18]. Therefore, cancer awareness and education efforts directed at PLHIV that takes advantage of their more frequent contact with the health care systems are needed to mitigate their higher cancer risk due to HIV infection [19].

Assessment of the knowledge, attitudes and practices of malignancies among PLHIV is an essential first step to determining the most effective approach to cancer education in this at-risk population. In this study, we used focus group discussions and key informant interviews to elucidate information on the level of awareness, attitudes and practices of cancer among PLHIV and the health care professionals who care for them in Nigeria. The level of awareness of cancer among the general population in Nigeria is low [18,20] and we hypothesized that it will be low among PLHIV particularly about those cancers that are more common in this population and less so in the general population [21-25].

## Materials and methods

We conducted a multi-site study at 4 health institutions that offer HIV care and treatment services in Nigeria - the University of Benin Teaching Hospital (UBTH) and the Nnamdi Azikiwe University Teaching Hospital (NAUTH) located in southern Nigeria, and the Federal Medical Centre Keffi (FMC Keffi) and the University of Abuja Teaching Hospital (UATH) in North-Central Nigeria. These sites where selected because they are hospitals where the Institute of Human Virology Nigeria provides PEPFAR services. They have the highest volume of clients accessing care and treatment of all the IHVN supported hospitals and we expected that we would get a wider variety of participants from these hospitals. In addition, to make this study more nationally relevant, we selected 2 hospitals from the North of the country and 2 from the south to ensure an even geographic spread.

We conducted 8 focus group discussions (FGD) (2 per institution) to determine the knowledge, attitudes and practices of PLHIV and non-PLHIV about AIDS associated Malignancies. We randomly selected PLHIV participants using a computer generated list of random numbers from the Institute of Human Virology (IHVN) database of HIV+ clients receiving care and treatment at the 4 hospitals. The HIV- participants were selected from patients or relatives of patients who were at the General Outpatient Department at the selected hospitals on the date of the focus group discussions. These persons were approached and invited to participate in a discussion on a pertinent health problem and were not told before-hand that this discussion would be about cancer. Recruitment continued until there was a group of ten people for each of the two groups at each site. Written informed consent was obtained from all individuals who were willing to participate in the study. FGDs were conducted separately for HIV+ and HIV- groups of 10 persons. The groups were heterogeneous and contained both male and female participants in each group. Participants had a wide variety of educational backgrounds from primary to tertiary education. The discussions lasted approximately 45–60 minutes, were managed by a researcher and a note-taker and were carried out in English language.

We also conducted Key Informant Interviews (KII) with 8 HIV care providers, 2 persons per institution - one doctor and one nurse - directly involved in the management of PLHIV. The participants for the KII were selected by visiting the Special Care and Treatment Clinic (STC) and interviewing the doctors and nurses that had run the clinic and seen patients on the day the FGD were carried out.

Data was audio recorded, and recordings were transcribed by the study staff. The data was transcribed by a member of the team who did not partake in carrying out the focus group discussions or key informant interviews. Handwritten notes taken during the interviews were used to supplements the audio recordings. Analysis was done using a thematic analysis. First we identified the themes and subthemes using both *a priori* and inductive approaches. Then we selected the themes most important to the study. Recurring themes were identified and grouped according to thematic areas. Comments were identified as recurring if two or more participants gave the same response. We subsequently developed a coding scheme using the open coding method by extracting major themes expressed by the participants and then coding sentences according to the various themes. Themes were analyzed for each question within individual focus group session as well as across the eight group sessions. Themes and conclusions were reviewed to ensure that the data was accurately reflected.

The FGD guide was developed by both authors based on the literature and according to the following domains: (1) Knowledge of cancer, its causes and knowledge of AIDS associated malignancies, (2) Attitude towards cancer risk, cancer screening, cancer diagnosis and treatment (3) Practices of cancer screening. We asked the participants open-ended questions about their knowledge of cancer, how they heard about cancer, what they think causes cancer, if they think they are at risk of cancer and if cancer can be treated. We also asked if they had ever been screened for cancer and their perceptions about why patients with cancer present late to hospitals. For the Key Informant Interviews, the guide contained domains on knowledge of cancer, cancer risk perception, attitude towards patients with cancer, knowledge of cancer screening, perception of PLHIV attitudes towards cancer screening, cancer diagnosis and treatment. For the purpose of this manuscript, the analysis and results focus on the following three major themes:

● Knowledge of cancer

● Attitudes towards cancer risk

● Practices of cancer screening

Quotations that best illustrated the themes of interest were selected and included in the manuscript. Data from the FGDs were transcribed with no unique identifiers or names used in the transcripts. The audio recordings were kept in a password protected laptop. Participants were assured of confidentiality of their discussions before they signed the informed consent forms.

The demographic data of the study participants including gender, marital status and educational status were collected and are presented in a tabular format in our results. When the focus group discussions were over, participants were given 500 Nigerian Naira ($3.50) to thank them for their time and contribute to their transportation costs. This study was approved the Institute of Human Virology Nigeria’s Ethics Committee.

## Results

### FGD Sample description

There were 80 participants, 20 from each of the 4 participating hospital sites. The participants’ ages ranged from 18 to 65 years with mean (SD) of 38 (2.8) years. Majority of FGD participants were women (60%). Most, 64% of participants had attained at least secondary level education, 30% had tertiary education and 6% had only primary level education. About two-thirds of the participants were married (66%), 24% were single, 5% were separated and 5% were co-habiting. (Table 1)

**Table 1:** Description of PLHIV and non-PLHIV FGD participants

	**No. of PLHIV participants**	**No. of Non-PLHIV participants**	**Total no. in both PLHIV and non-PLHIV (% of total)**
**Gender**			
Male	18	14	32 (40)
Female	22	26	48 (60)
Total	40	40	80 (100)
**Educational Status**			
Primary level	431	1	5(6)
Secondary level (high schoolequivalent		20	51(64)
Tertiary level	5	19	24(30)
Total	40	100	100
**Marital Status**			
Married	30	23	53(66)
Single	8	11	19(24)
Separated/widowed	4	4	4(5)
Co-habiting	2	2	4(5)
Total	40	40	80(100)

### Knowledge of cancer

Most, (80%) participants had heard about cancer and considered it a fatal disease but displayed poor knowledge of its causes. (Table 2) Knowledge about AIDS defining cancers such as Cervical Cancer, Kaposi Sarcoma (KS) and Non-Hodgkin’s Lymphoma (NHL) were particularly poor although most had heard about breast cancer. Participants identified electronic media, newspapers, and health talks at hospitals as the medium through which they had heard about cancer. Most respondents did not believe that it is possible to have HIV and cancer though some opined that it may be possible since both were caused by viruses. A few of them believed that cancer can be treated if caught early but most were of the opinion that cancer is a deadly disease that has no cure.

**Table 2:** Knowledge of Cancer and its causes among PLHIV and non-PLHIV in Nigerian tertiary health institutions

**Knowledge**	**Substantial finding**	**Illustrative Quote**
Knowledge of cancer	Poor knowledge of cancer	“Cancer is a deadly disease that has no cure” non-PLHIV respondent
		“I believe it is a disease that is very rare in men. I have not heard of any man who has had cancer and died but women die of breast cancer” PLHIV respondent
Causes of cancer	Limited knowledge of causes of cancer	“I have heard that body creams cause cancer and also women who put money in their bra (underwear) fit get cancer” PLHIV participant
		“If one drinks dirty water from a dirty bowl or cup that is rusted, one can get cancer” Non PLHIV
		“Neglect can cause cancer, if an old person is neglected by their family, they can get cancer” PLHIV respondent
Symptoms of cancer	Symptoms of breast cancer most commonly remembered	“Someone with cancer can have fever, pain and breast lump” Non PLHIV respondent
Knowledge of HIV-associated cancers (CC, KS and NHL)	Limited knowledge of HIV –associated cancers among PLHIV	“I have no idea if HIV and Cancer are related” PLHIV respondent
		“Kaposi what? Which one is that one again, me I never hear oh” (laughs) PLHIV respondent. sic- (Kaposi what? what does that mean? I have never heard of it).
		“Cervical cancer is mainly on women on their private part” PLHIV respondent
Treatment for cancer	Poor knowledge of cancer treatment	“Native leaves can also cure cancer, like back of mango fruit and guava fruit” PLHIV respondent -(traditional leaves can cure cancer, like leaves from mango fruit and guava fruit)
	Misconceptions about cancer treatment	“Herbalist can cure cancer” non-PLHIV respondent
		“I heard that one can have operation and medicine to cure cancer if caught early” PLH V respondent sic-(I heard that one can have surgery and medications to cure cancer if caught early)

The knowledge of cancers was similar among PLHIV and those who were not living with HIV/AIDS. However, there were differences in the level of knowledge comparing FGD participants in the institutions in northern part of the country with those in the southern part. Participants in the south were more knowledgeable about cancer and AIDS associated cancers than those in the north. No participants from the 2 hospitals in the north had ever heard about cervical cancer, Kaposi sarcoma or Non- Hodgkin’s lymphoma. However, some participants from the south who had attained tertiary education specifically mentioned Kaposi sarcoma as an AIDS associated cancer while another said, “I heard that lymphoma at the neck can be seen in people with HIV.” Women also appeared more knowledgeable about cancer than men.

### Attitudes towards cancer/cancer risk

FGD participants admitted to being ‘very scared’ whenever the word cancer was mentioned (Table 3). Many did not believe that they could develop cancer. One of the PLHIV participants voicing her disbelief said *‘it is not possible for me to get cancer, God will not allow it’*. They were also concerned about the cost of treatment. (Table 3) Participants considered cancer to be a fatal condition, one which has no treatment except through “supernatural” intervention. At least one of the participants opined that the ‘*endpoint (of all cancer diagnosis) is death*’ as shown in Table 3. Some participants suggested that their strong faith in God and their spirituality would ensure that God will cure them should they ever have cancer. (Table 3) Some study participants said that they or their relatives would prefer to seek alternate medicine practitioners if they had cancer. They explained that the reason for their patronage of alternative medicine practitioners is because these practitioners were better at keeping their diagnosis or treatment secret. There was no difference in attitudes towards cancer between the PLHIV and the non-PLHIV but more women than men were of the opinion that God would not ‘allow’ them get cancer. More participants in the south of the country also demonstrated greater belief than those in the north that God is ultimately the one to decide who gets cancer.

**Table 3:** Attitudes towards cancer risk, diagnosis, and treatment among PLHIV and non-PLHIV in Nigerian tertiary health institutions

**Attitude**	**Substantial finding**	**Illustrative quotes**
Attitude towards cancer diagnosis	Fear	“Once I hear cancer I feel very scared” PLWA respondent
Attitude towards risk of cancer among PLHIV	Disbelief	“It is not possible that someone who has HIV can have cancer, I do not believe it” PLHIV respondent
Attitude towards cancer treatment	Fatalism	“The endpoint of cancer is death,” non-PLHIV respondent
	Denial	“It is only God who is supernatural that can treat this disease” non-PLHIV respondent
Attitude towards individual cancer risk		“.me I no fit get cancer for my body” PLHIV respondent. sic- (I cannot get cancer)

### Practices of cancer screening

According to the FGD participants, many people, including the FGD participants, were aware that there is screening for cancer (Table 4) but only few of the FGD participants had actually been screened. They attributed this low level of screening uptake to lack of knowledge about the benefits of screening and the belief that it is better to be ignorant of a diagnosis of cancer if one has no symptoms of the disease as screening with resultant diagnosis of cancer can make you ‘die early’. The participants associated screening with early mortality due to emotional distress arising from cancer diagnosis. Although the participants understood screening to mean testing for a particular disease, of the 3 participants who had been screened for cervical cancer of the 48 eligible women, one said the result was not disclosed to her while the other 2 said they had negative results. Only 6% of those eligible for cervical cancer screening had ever been screened. (3 of 48 female participants). Major concerns raised by the participants included cost of screening and fear of a false positive diagnosis expressed particularly by a female participant who said, ‘*I know someone who was wrongly diagnosed of HIV it can also happen with cancer, mistakes can happen*’. FGD participants with low levels of education in the northern part of the country were concerned that screening is a means of ‘inviting cancer to one’s body’ suggesting that screening is associated with increased risk of cancer.

**Table 4:** Practices of cancer screening among PLHIV and non-PLHIV attending clinics in Nigerian tertiary hospitals

**Practices of cancer screening**	**Substantial finding**	**Illustrative quotes**
Knowledge of screening	Participants were fairly knowledgeable of what screening for cancer means.	“Screening is where the professionals check you to know if you have cancer or not” PLHIV respondent
Reasons for poor screening practices	Fear	“They don’t want to do screening for cancer because it is like asking God to give you the disease; it is like inviting cancer to your body” non-PLHIV respondent
	Cost	“If the screening is free I will like to do it, the hospital where I go to the screening for men is4000 naira, it is too expensive” PLHIV respondent
	Use of alternative health care	“Some people do not want to go and do the test because they are afraid that their secret will come out, it is better to go to the herbalist where you will not meet many people and the secret will be kept” PLHIV respondent
General screening acceptability	Participants expressed willingness to participate in screening	“I will participate in any screening test in the hospital because I want to know if I have cancer or not” non-PLHIV respondent
Previous history of screening	Only 3 participants in our study had ever been screened for cancer.	“I was screened in the clinic for cervical cancer, it was free. PLHIV respondent
Recommendations to improve cancer information and cancer screening practices	Participants mentioned mass and electronic media, mobile text messaging, health talks and campaigns as useful methods of disseminating information.	“They can give regular health talks at the hospitals and offer free screening” PLHIV respondent
		“You can go to different sport centers where youths gather to watch football and spread the information” non-PLHIV respondent

### Key informant interviews

We interviewed 8 health care professionals (4 doctors and 4 nurses) who provide care for PLHIV at the 4 study sites.

### Knowledge of cancer and AIDS associated cancers

Findings from the KII showed that although health care professionals particularly doctors were aware of cancer and had good knowledge of its causes, they rarely discussed cancer in general or AIDS associated cancers with HIV patients during clinic visits. The health care professionals identified the causes of cancer to be smoking, past family history of cancer and infectious agents. They also mentioned skin and leg sores, vaginal bleeding, and neck swelling be suspicious symptoms in PLHIV. Majority of the health professionals were of the opinion that most PLHIV lack basic knowledge about cancer and its causes and they identified the need for cancer education in the care and treatment of PLHIV.

"“PLHIV need to be provided with more information on cancer and its causes, it is true that we do not routinely provide this information in the clinics as a result of high caseload and lack of time”. Physician"

### Attitudes

There was general agreement that a diagnosis of cancer is associated with fear. However, majority of the KII respondents believe that cancer can be treated, if caught early. One of the nurses mentioned that most times even after treatment, cancer patients often die. Discussants thought that cancer is becoming more common than in the past and that even they as health workers could possibly be diagnosed of cancer.

"‘Even as a health worker I am not immune to cancer, anyone can get cancer, the important thing is to catch it early.’ Nurse"

Health care professionals mentioned that surgery and chemotherapy were the most commonly used methods of treatment for cancer in their institutions. There was a general agreement that there is need to increase awareness of cancer among not only PLHIV but the general population.

### Cancer screening practices

All the health care professionals interviewed had heard of cancer screening, and were of the opinion that screening is a necessary component of clinical care for the general population and for PLHIV. Of the 8 health care professional interviewed of which 5 were women and eligible for screening, 2 (40%) had been screened for cancer (cervical cancer) as opposed to. When the issue of cancer screening in PLHIV was raised, they identified high cost as a major deterrent to cancer screening in PLHIV. One of the health care professionals was of the opinion that if screening is done at minimal/no cost, there would be an increase in screening uptake in Nigeria. She opined that “*cost is a major barrier to screening uptake in Nigeria; it is just too expensive especially for people who are looking for money to pay transportation costs to the hospital, where do they now get the extra money to pay for screening*”.

When asked if they think that HIV positive patients can get cancer, they all agreed that this was possible. According to a medical doctor interviewed, *“PLHIV just like the general population are at risk of cancer and perhaps a little more because of the additional risk HIV infection confers”*. However the health professionals mentioned that cancer risk is not a topic that is routinely discussed with PLHIV at clinic visits and PLHIV are not routinely screened for cancer.

Mass media campaigns, television and radio adverts, subsidized screening programs and health talks were identified by the respondents as useful ways of disseminating information on cancer to PLHIV and the general population.

## Discussion

PLHIV have a higher risk of malignancies than the general population [19,26,27]. Therefore, we conducted FGD and KII with the primary objective of evaluating the knowledge, attitudes and practices of malignancies among people living with HIV. In this study, we found that most of our FGD participants had heard about cancer but had limited knowledge particularly of AIDS-associated malignancies, causes of cancer and availability of treatment. The participants expressed strong views on the association between HIV and cancer with most believing that it is not possible to develop cancer if one was already infected with HIV. Some of the participants in the study expressed preference for alternative and traditional means of diagnosis and treatment. Very few participants in our study had ever been screened for any cancer despite knowledge that screening for cancer exists.

Findings from our study suggest poor knowledge of cancer and its causes among the FGD participants. This was surprising because we expected that PLHIV would be better informed about cancers particularly those associated with HIV given their more frequent contact with the health care system. Our results suggest the need for cancer education even in cohorts with high levels of interaction with the health care system such as PLHIV.

We observed that the attitudes of PLHIV in Nigeria towards cancer were characterized by fear and low level of perceived cancer risk. Majority of our participants had heard about cancer screening, but very few had ever been screened for cancer. Similarly low levels of cancer screening had been reported in southeastern Nigeria, although in a different target population [28]. Participants in our study identified fear of diagnosis, fear of a false positive diagnosis and cost as the primary barriers to the uptake of cancer screening among PLHIV in Nigeria.

Most FGD participants who said they or their relatives patronize alternate medical practitioners were those who had attained secondary education or less. The literature identifies poverty and lack of education as the reasons for high patronage of complementary and alternative medicine practitioners [17]. PLHIV in our study mentioned that they patronize alternate medical practitioners because these practitioners are better at keeping patients’ diagnosis and treatment secret. This finding can be interpreted to mean that there is a level of distrust that PLHIV associate with hospitals and health care professionals. It is also possible that PLHIV are concerned about the complexity of services and personnel that they interact with in the hospital and they believe that this increases the risk of inadvertent disclosure in contrast to alternative medicine practitioners which are often one person establishments.

With the recent attention paid to HIV/AIDS in Nigeria, awareness of HIV/AIDS has substantially increased, however, cancer education and awareness remains poor [29]. More than 70% of all cancer patients in Nigeria present with advanced disease [23], therefore opportunities to incorporate cancer screening into routine HIV care can play a pivotal role in reducing the cancer burden among PLHIV [21,29,30]. HIV clinics have expanded their operations to incorporate treatment of opportunistic infections such as tuberculosis and they can similarly incorporate cancer prevention, early diagnosis and treatment services. This model has been successfully demonstrated by programs in Zambia and other parts of Africa with resultant saving of lives [31,32].

Health care professionals including those caring for PLHIV need to be trained to incorporate cancer prevention and education services, and recognize the early signs and symptoms of cancer, particularly those prevalent among PLHIV. When coupled with coordinated referral system, this can optimize the prevention and management of cancer among PLHIV. Such efforts are likely to be more effective if complemented by cancer education programs for the HIV/AIDS patient population so they can appreciate and take advantage of cancer prevention services. Such cancer education can be delivered through electronic and print media, mobile text messages, campaigns and other health and educational programs as suggested by our FGD participants.

Our findings are important because they show low levels of cancer awareness among PLHIV despite their high level of interaction with the health care system for HIV treatment and prevention. This is similar to findings from other studies that there is low level of cancer awareness in Nigeria [28]. While the health care professionals who participated in our KII were fairly knowledgeable of cancer and AIDS associated cancers, the low levels of awareness in the population they serve suggest that this knowledge does not translate into screening, early detection and timely referral of cancer patients. Our results demonstrate a need for clients’ and health professionals’ education to promote early detection of cancer and increased use of cancer prevention services.

Furthermore, successful implementation of cancer prevention and control strategies among PLHIV will require a commitment by relevant government agencies to the development of appropriate policies and guidelines to support extra resources for community health education and awareness, infrastructure development, training of health care personnel on early detection and diagnosis of AIDS associated malignancies, subsidized cancer screening interventions and the evaluation of cancer prevention strategies through the promotion of research and cancer surveillance. Provision of well-equipped pathology laboratories for histologic examination of suspected cases of cancer is also important.

Our study provides valuable information about current knowledge, attitude and awareness of cancer among PLHIV in Nigeria. It is however limited by the small sample size and the use of qualitative research methods. Nevertheless, qualitative methods are appropriate where researchers need to probe for information that may be unstructured and not amenable to survey methods. It is also possible that the social interaction necessitated by the FGD methods created an atmosphere where perceived socially desirable responses had been given. Some participants’ responses may have been influenced by those of more vocal participants. However, we ensured that quieter participants in the FGD had an opportunity share their thoughts and contribute to the discussion. In order to improve the generalizability of our results, we randomly selected participants from high volume HIV/AIDS centers in Nigeria and we believe that their opinions were a true reflection of the situation among PLHIV in Nigeria.

## Conclusion

Our study shows that there is poor knowledge of cancer and its causes among PLHIV in Nigeria and that there is need to develop appropriate health education strategies to promote cancer prevention, screening, and management. These can incorporated into clinic visits for PLHIV and training should be provided for health care professionals on early diagnosis of AIDS-associated cancers. Furthermore, cancer control policies and guidelines for PLHIV should be developed and implemented by relevant donor and government agencies.

## Competing interest

The authors declare no conflict of interest.

## Authors’ contributions

Both authors contributed equally to the writing of this paper. All authors read and approved the final manuscript.
